# Two novel fomannosane-type sesquiterpenoids from the culture of the basidiomycete *Agrocybe salicacola*

**DOI:** 10.1007/s13659-012-0031-2

**Published:** 2012-05-18

**Authors:** Liang-Yan Liu, Zheng-Hui Li, Ze-Jun Dong, Xing-Yao Li, Jia Su, Yan Li, Ji-Kai Liu

**Affiliations:** 1 State Key Laboratory of Phytochemistry and Plant Resources in West China, Kunming Institute of Botany, Chinese Academy of Sciences, Kunming, 650201 China; 2 Graduate University of Chinese Academy of Sciences, Beijing, 100049 China

**Keywords:** *Agrocybe salicacola*, fomannosane-type sesquiterpenoids, agrocybins

## Abstract

Two novel fomannosane-type sesquiterpenoids, agrocybins H (**1**) and I (**2**), together with a known compound illudosin (**3**), were isolated from the culture broth of the mushroom *Agrocybe salicacola.* Their structures were elucidated by extensive spectroscopic analysis. The relative stereochemistry of **1** was determined by the use of single crystal X-ray crystallographic diffraction.

**Electronic Supplementary Material:**

Supplementary material is available for this article at 10.1007/s13659-012-0031-2 and is accessible for authorized users.

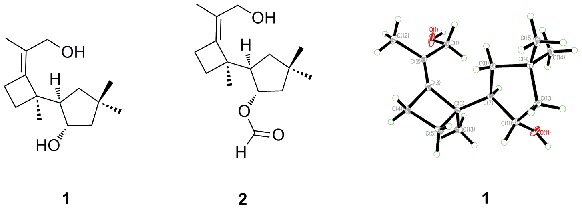

## Introduction

Fomannosane-type sesquiterpenoids (FS) were biogenetically belonging to the largest group of sesquiteroenes derived from humulene, a monocyclic C-15 hydrocarbon.[1,2] The typical feature of FS is the existence of a cyclobutane and a cyclopentane connecting by the C–C bond.[2,3] So far, fewer than ten compounds of this kind have been reported with anti-bacterial, antimicrobial, and cytotoxic activities.[3–6] The fungus *Agrocybe salicacola* (Chinese name, Yang-Liu-Tian-Tou-Gu) is a delicate edible mushroom endemic to Yunnan province, China, which grows at trunks of poplars and willows from September to October.[7,8] In the previous study, seven new sesquiterpene compounds, together with three known components, were isolated from the culture broth of *A. salicacola*.[9] In a continuing investigation of this fungus, two novel fomannosane-type sesquiterpenoids, agrocybin H and I (**1** and **2**), together with the known illudosin (**3**)[10], were obtained. The structures of compounds **1** and **2** were determined on the basis of extensive spectroscopic analysis and the X-ray crystallographic diffraction.

## Results and Discussion

Compound **1**, colorless needles, was assigned the molecular formula $C_{15}H_{26}O_2$ on the basis of the positive HRESIMS at *m/z* 261.1833 ([M + Na]$^+$), required three degrees of unsaturation. Inspection of the $^{13}$C NMR spectrum revealed the existence of fifteen carbon signals, including four methyls, five methylene groups (one oxymethylene), two methines (one oxygenated), and four quarternary carbons (including two $sp^2$ ones). These

**1** R = H
**2** R = CHO

**3**

data suggested that **1** was a bicyclic sesquiterpenoid. Ring A was elucidated on the basis of $^1$H-$^1$H COSY correlations of $\delta_H$ 2.23 (1H, m, H-7) with $\delta_H$ 1.59 (1H, dd, $J$ = 12.3, 6.4 Hz, H-8a), 1.24 (1H, t, $J$ = 12.3 Hz, H-8b), and 3.89 (1H, m H-11), and of H-11 with H-10 [$\delta_H$ 1.72 (1H, dd, $J$ = 13.4, 7.9 Hz, H-10a), and 1.49 (1H, dd, $J$ = 13.4, 4.1 Hz, H-10b)], coupled with HMBC correlations from $\delta_H$ 1.10 (3H, s, H-14) and 1.00 (3H, s, H-15) to $\delta_C$ 37.3 (s, C-9), 44.6 (t, C-8), and 52.3 (t, C-10) (Figure 1). Ring B was determined to be a four-membered ring, as supported by the $^1$H-$^1$H COSY and HMBC spectra. Specifically, the proton signals at $\delta_H$ 2.50 (1H, m, H-4a) and 2.33 (1H, m, H-4b) correlated with signals at $\delta_H$ 1.87 (1H, ddd, $J$ = 10.9, 10.8, 6.2 Hz, H-5a) and 1.56 (1H, ddd, J = 10.9, 10.2, 6.3 Hz, H-5b) in the $^1$H-$^1$H COSY spectrum; moreover, H-4a and H-4b showed HMBC correlations with $\delta_C$ 49.6 (s, C-6), 127.1 (s, C-2), and 144.9 (s, C-3), and H-5a and H-5b showed HMBC correlations with C-3 and C-6. Furthermore, the HMBC correlations from the proton at $\delta_H$ 2.23 (1H, m, H-7) to $\delta_C$ 49.6 (s, C-6), 26.5 (t, C-5), and 144.9 (s, C-3) revealed that ring A connected with ring B by C-6 and C-7 as shown in Figure 1. The X-ray crystallographic diffraction analysis

*To whom correspondence should be addressed. E-mail: jkliu@mail.kib.ac.cn

**Figure 1.** Key 2D NMR correlations of compounds **1** and **2**

confirmed the above elucidation, and determined the relative stereoconfiguration of **1** (Figure 2).

Compound **2** was obtained as colorless oil. The positive HRESIMS showed a pseudo molecular ion at *m/z* 289.1772 ([M + Na]$^+$), corresponding to the formula $C_{16}H_{26}O_3$ that required four degrees of unsaturation. The 1D NMR data of **2** were highly similar with those of **1** (Table 1), except for the additional formyl group in **2**. In the HMBC spectrum, the formyl group at $\delta_H$ 8.08 (1H, s) showed a strong correlation with $\delta_C$ 77.9 (d, C-11) (Figure 1), suggesting that **2** is the 11-*O*-formyl derivative of **1**.

The known compound illudosin (**3**) had been reported previously by Arnone et al.,[10] whose absolute stereochemistry was determined by 1D and 2D NMR experiments and exciton chirality method. Illudosin was supposed to be an intermediate in the biosynthesis of the susquiterpene fomannosin.

Compounds **1**–**3** were tested for their cytotoxicity against five human cancer cell lines (HL-60, SMMC-7712, A-549, MCF-7, and SW480) by the MTT method, with DDP and taxol as positive controls. None of the compounds exhibited obvious activity at the concentration of 40 $\mu$M.

**Figure 2.** The X-ray stereoconfiguration of compound **1**

## Experimental Section

**General Experimental Procedures.** Melting point was surveyed with an X-4 microscopic melting point meter. Optical rotations were measured on a Horiba SEPA-300 spectropolarimeter. UV spectra were recorded on a Shimadzu double-beam 210A spectrometer. IR spectra were obtained on a Bruker Tensor 27 FT-IR spectrometer using KBr pellets. NMR spectra were acquired on Bruker DRX-500 and AM-400 instruments at room temperature with TMS as an internal standard. Chemical shifts ($\delta$) were expressed in ppm with reference to the solvent signals. Mass spectra (MS) were recorded on an API QSTAR time-of-flight spectrometer or a VG Autospec-3000 spectrometer. X-ray crystallographic data were collected on a Bruker APEX DUO diffractometer with graphite-monochromated Mo K$\alpha$ radiation. Silica gel (200–300 mesh, Qingdao Marine Chemical Inc., China), Sephadex LH-20 (Amersham Biosciences, Sweden), and RP-18 gel (40–75 $\mu$m, Fuji Silysia Chemical Ltd. Japan) were used for column chromatography (CC). Preparative HPLC (Prep-HPLC) was performed on an Agilent 1100 liquid chromatography system equipped with a Zorbax SB-$C_{18}$ column (9.4 mm × 150 mm). Pre-coated silica gel GF254 plates (Qingdao Marine Chemical Inc., China) were used for TLC. Fractions were monitored by TLC, and spots were visualized by heating silica gel plates sprayed with 10% $H_2SO_4$ in ethanol.

**Fungal Material and Cultivation Conditions.** The fungus *A. salicacola* was collected at the Botanic Garden of Kunming Institute of Botany, Chinese Academy of Sciences, China, in spring 2008, and identified by Prof. Mu Zang, Kunming Institute of Botany. The voucher specimen has been deposited in the Herbarium of Kunming Institute of Botany, Chinese Academy of Sciences. The liquid culture medium contained saccharine 5%, yeast powder 0.5%, peptone 0.15%, $KH_2PO_4$ 0.05%, and $MgSO_4$ 0.05%. Inoculums of *A. salicacola* were prepared in a 15L-fermentor (Biostar, Shanghai Guo Qiang, China) for 6 days under the following conditions: culture temperature 24 °C, initial pH 6.0, agitation speed 250 r/min, inoculation volume 10% (by volume), and aeration rate 1.0 vvm. Then, the liquid seed was transferred into a 100L-fermentation tank to be cultivated under the same conditions for 20 days to afford 80 L culture broth.

**Extraction and Isolation.** The entire culture broth of *A. salicacola* (80 L) was initially filtered, and the filtrate was extracted three times with EtOAc. The organic layer was concentrated under reduced pressure to give a crude extract (280 g), and further subjected to Column Chromatography (CC) over silica gel using a petroleum ether-$Me_2CO$ gradient (1:0 → 0:1) to afford fractions A–L. Fraction F (65 g) was loaded on CC over silica gel to provide four subfractions ($F_1$–$F_4$). Fraction $F_2$ was first separated by silica gel CC (petroleum ether-$Me_2CO$, 5:1), then purified by Prep-HPLC (MeCN/$H_2O$, 2:8 → 5:5) to afford compound **2** (2 mg). Compound **1** (38 mg) was obtained from fraction $F_4$ by repeated column chromatography over silica gel. Fraction H was performed on silica gel CC eluted using a chloroform-methanol gradient (10:1 → 1:1), and then purified by Sephadex LH-20 (chloroform:methanol 1:1) to afford compound **3** (5 mg).

**Agrocybin H (1):** colorless needles (acetone); mp 141–142 °C; $[\alpha]_D^{17}$ + 88.2 (*c* 0.22, MeOH); UV (MeOH) $\lambda_{max}$(log $\varepsilon$) 203 (3.41) nm; IR (KBr) $\nu_{max}$ 3248, 2949, 2925 cm$^{-1}$; $^{13}$C and $^1$H NMR data, see Table 1; ESIMS (positive) *m/z* 261 [M + Na]$^+$; HRESIMS (positive) *m/z* 261.1833 (calcd. for $C_{15}H_{26}O_2Na$, 261.1830).

**Agrocybin I (2):** colorless oil; $[\alpha]_D^{13}$ + 19.9 (*c* 0.20, MeOH); $^{13}$C and $^1$H NMR data, see Table 1; ESIMS (positive) *m/z* 289

**Table 1.** **$^{1}$H (400 MHz) and $^{13}$C (100 MHz) NMR spectroscopic data for compounds 1 and 2**

| position | **1**[a] | | **2**[b] | |
|---|---|---|---|---|
| | $\delta_H$ | $\delta_C$ | $\delta_H$ | $\delta_C$ |
| 1 | 4.03, d (11.6); 3.95, d (11.6) | 62.9, $CH_2$ | 3.98, dd (12.3, 5.3) | 62.5, $CH_2$ |
| 2 | | 127.1, C | | 128.2, C |
| 3 | | 144.9, C | | 142.0, C |
| 4 | 2.50, m; 2.33, m | 24.6, $CH_2$ | 2.48, m; 2.32, m | 24.2, $CH_2$ |
| 5 | 1.87, ddd (10.9, 10.8, 6.2); 1.56, ddd (10.9, 10.2, 6.3) | 26.5, $CH_2$ | 1.91, m; 1.56, m | 25.8, $CH_2$ |
| 6 | | 49.6, C | | 48.6, C |
| 7 | 2.23, m | 56.1, CH | 2.59, m | 53.1, CH |
| 8 | 1.59, dd (12.3, 6.4); 1.24, t (12.3) | 44.6, $CH_2$ | 1.85, dd (14.3, 8.2); 1.50, overlaped | 49.6, $CH_2$ |
| 9 | | 37.3, C | | 38.0, C |
| 10 | 1.72, dd (13.4, 7.9); 1.49, dd (13.4, 4.1) | 52.3, $CH_2$ | 1.70, dd (12.9, 7.9); 1.33, t (12.9) | 44.0, $CH_2$ |
| 11 | 3.89, m | 75.9, CH | 4.95, m | 77.9, CH |
| 12 | 1.52, s | 14.8, $CH_3$ | 1.51, s | 14.9, $CH_3$ |
| 13 | 1.35, s | 27.2, $CH_3$ | 1.23, s | 27.3, $CH_3$ |
| 14 | 1.10, s | 29.9, $CH_3$ | 1.08, s | 28.6, $CH_3$ |
| 15 | 1.00, s | 30.9, $CH_3$ | 1.04, s | 30.0, $CH_3$ |
| 1′ | | | 8.08, s | 161.7, CH |

[a]spectra were measured in methanol-$d_4$; [b]spectra were measured in acetone-$d_6$.

$[M + Na]^+$; HRESIMS (positive) $m/z$ 289.1772 (calcd. for $C_{16}H_{26}O_3Na$, 289.1779).

**Crystallographic Data of Agrocybin H (1):** $C_{30}H_{52}O_4$ ($C_{15}H_{26}O_2$ × 2); $M$ = 476.72; Orthorhombic; space group $P2_12_12$; $a$ = 13.4396(2) Å, $b$ = 23.1684(3) Å, $c$ = 9.27430(10) Å; $\alpha = \beta = \gamma = 90°$, $V$ = 2887.78(6) Å$^3$; $Z$ = 4; $\rho$ = 1.096 g·cm$^{-3}$; crystal dimensions 0.50 × 0.11 × 0.06 mm$^3$; Shelxs97 with a graphite monochromator; Mo K$\alpha$ radiation. The total number of reflections measured was 15344, of which 4961 were observed, $I > 2\sigma(I)$; $R_1$ = 0.0769, $wR_2$ = 0.2074. Crystallographic data for agrocybin H (**1**) has been deposited at the Cambridge Crystallographic Data Centre as deposition number CCDC 866717. Copies of the data can be obtained free of charge on application to CCDC, 12 Union Road, Cambridge CB2 1EZ, UK (Tel: +44 (0)1223 762911, e-mail: deposit@ccdc.cam.ac.uk).

**Cytotoxicity Assay.** The following human tumor cell lines were used: HL-60, SMMC-7712, A-549, MCF-7, and SW480. All the cells were cultured in RMPI-1640 or DMEM medium (Hyclone, Logan, UT), supplemented with 10% fetal bovine serum (Hyclone) at 37 °C in a humidified atmosphere with 5% $CO_2$. Cell viability was assessed by conducting colorimetric measurements of the amount of insoluble formazan formed in living cells based on the reduction of 3-(4,5-dimethylthiazol-2-yl)-2,5-diphenyltetrazolium bromide (MTT) (Sigma, St. Louis, MO). Briefly, 100 $\mu$L of adherent cells were seeded into each well of a 96-well cell culture plate and allowed to adhere for 12 h before drug addition, while suspended cells were seeded just before drug addition, both with an initial density of 1 × 10$^5$ cells/mL in 100 $\mu$L of medium. Each tumor cell line was exposed to the test compounds at various concentrations in triplicate for 48 h, with DDP and toxal as positive controls. After the incubation, MTT (100 $\mu$g) was added to each well, and the incubation continued for 4 h at 37 °C. The cells lysed with 200 $\mu$L SDS after removal of 100 $\mu$L of medium. The optical density of lysate was measured at 595 nm in a 96-well microtiter plate reader (Bio-Rad 680). The $IC_{50}$ value of each compound was calculated by Reed and Muench's method.[11]

## Electronic Supplementary Material

Supplementary material is available in the online version of this article at http://dx.doi.org/ 10.1007/s13659-012-0031-2 and is accessible for authorized users.

## Acknowledgments

This project was supported by the National Basic Research Program of China (973 Program, 2009CB522300), and the National Natural Sciences Foundation of China (30830113, U1132607).

**Open Access** This article is distributed under the terms of the Creative Commons Attribution License which permits any use, distribution, and reproduction in any medium, provided the original author(s) and source are credited.

## Electronic supplementary material

Supplementary material, approximately 864 KB.
