# Supplementary material for: Two novel fomannosane-type sesquiterpenoids from the culture of the basidiomycete Agrocybe salicacola
Source: Nat Prod Bioprospect. 2012 May 18;2(3):130–2. doi: 10.1007/s13659-012-0031-2 (PMC4131595; doi:10.1007/s13659-012-0031-2)

Electronic Supplementary Material

Two novel fomannosane-type sesquiterpenoids from the culture of the basidiomycete *Agrocybe salicacola*

Liang-Yan LIU,[a,b] Zheng-Hui LI,[a] Ze-Jun DONG,[a] Xing-Yao LI,[a,b] Jia SU,[a,b] Yan LI,[a] and Ji-Kai LIU[a,*]

[a]State Key Laboratory of Phytochemistry and Plant Resources in West China, Kunming Institute of Botany, Chinese Academy of Sciences, Kunming 650201, China

[b]Graduate University of Chinese Academy of Sciences, Beijing 100049, China

Received 16 April 2012; Accepted 8 May 2012

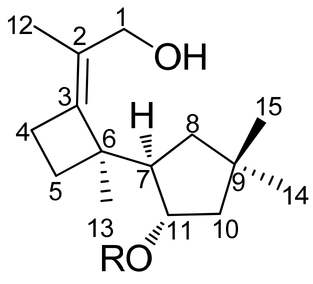

1 R = H
2 R = CHO

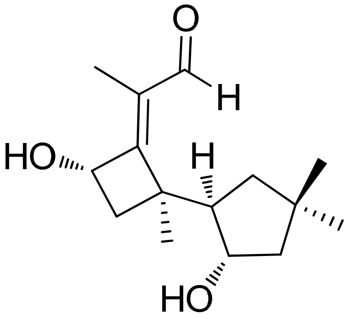

3

Structures of compounds **1**–**3**

Table of Contents

1D and 2D NMR spectra of agrocybin H **(1)** S2

1D and 2D NMR spectra of agrocybin I **(2)** S4

*To whom correspondence should be addressed. E-mail: jkliu@mail.kib.ac.cn

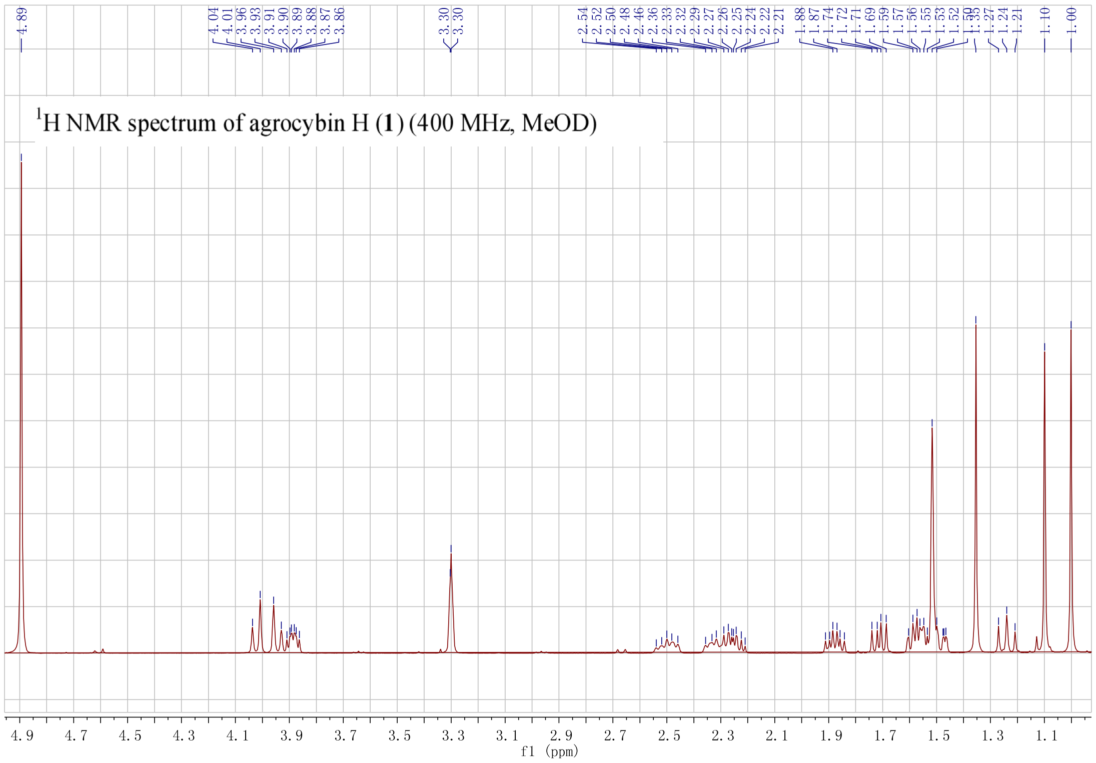

^{1}H NMR spectrum of agrocybin H (**1**) (400 MHz, MeOD)

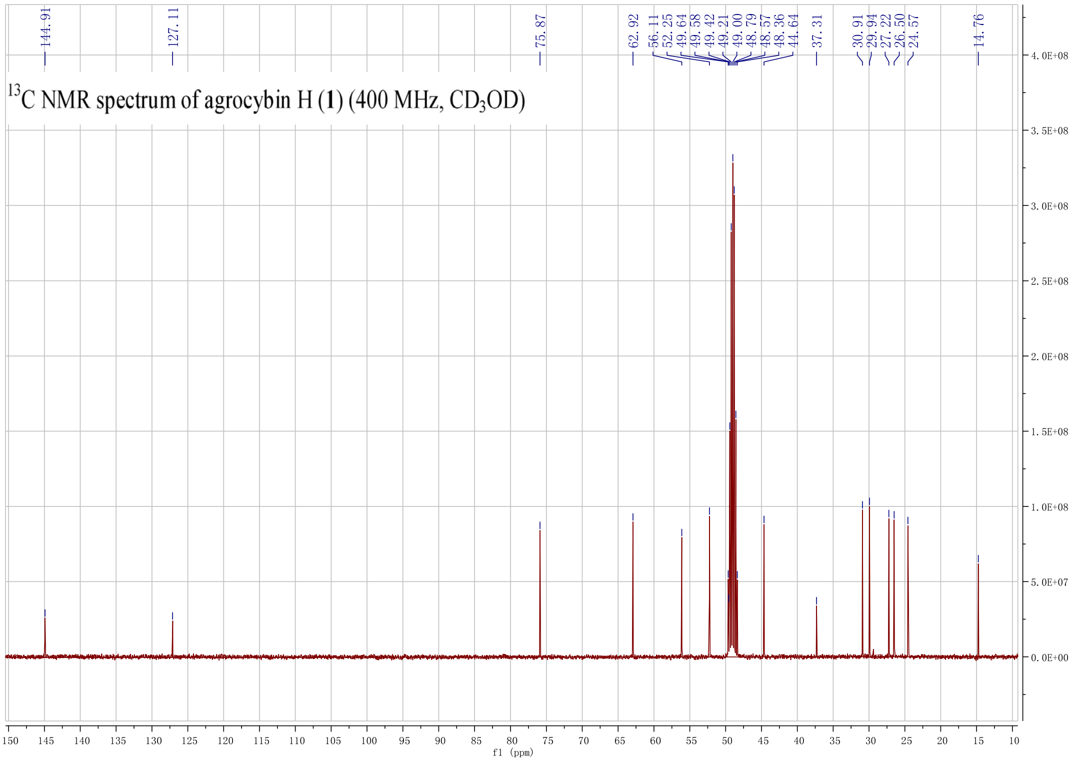

^{13}C NMR spectrum of agrocybin H (**1**) (400 MHz, CD_3OD)

HSQC spectrum of agrocybin H (1)

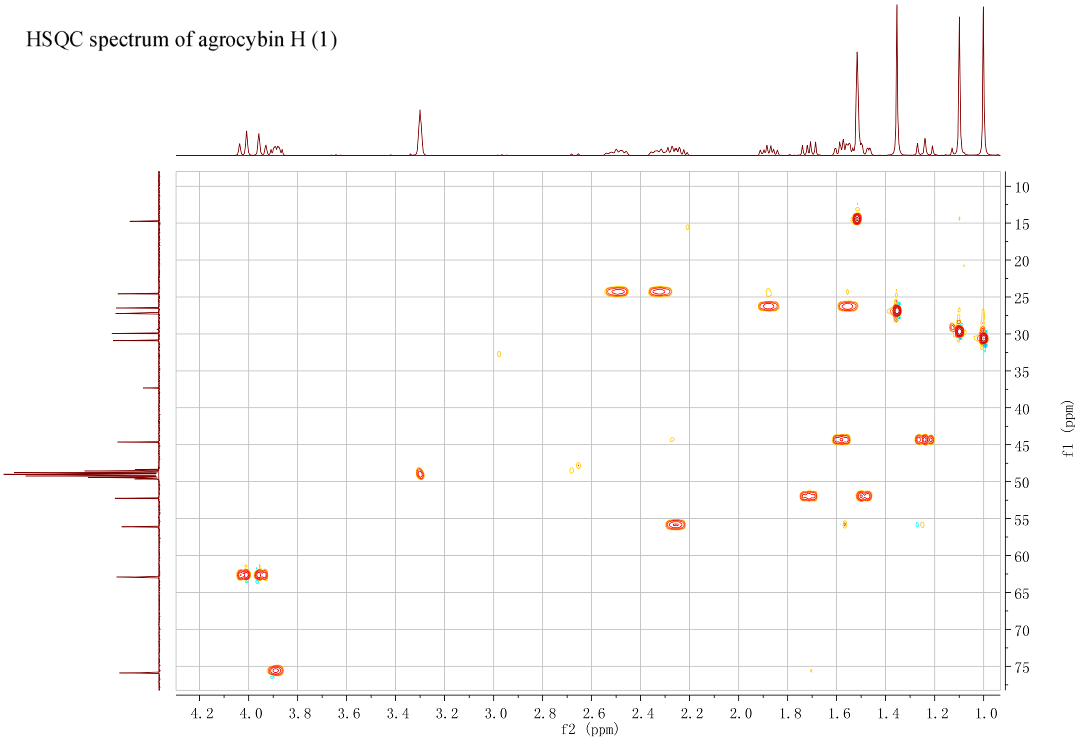

HMBC spectrum of agrocybin H (**1**)

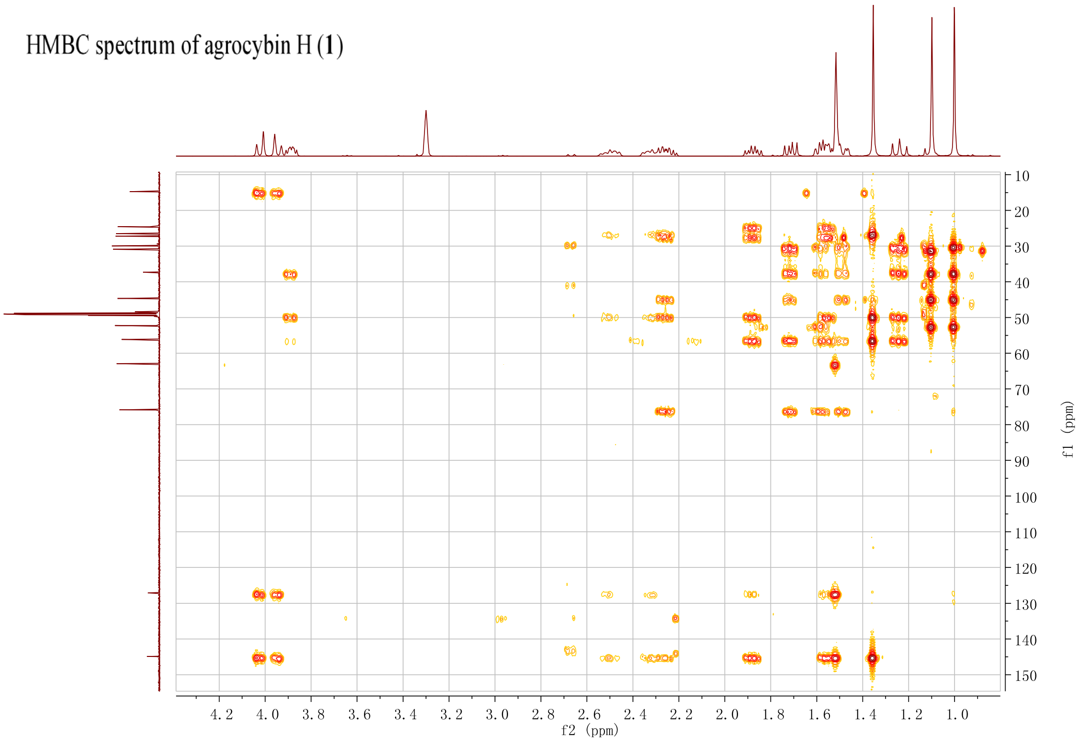

COSY spectrum of agrocybin H (**1**)

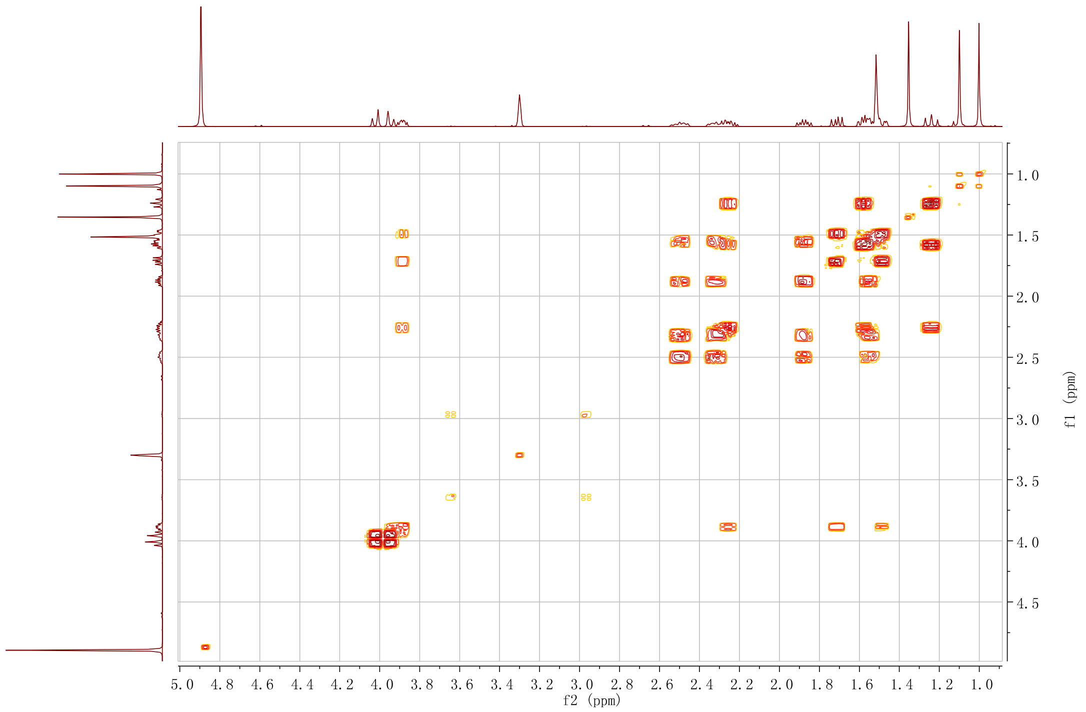

^{1}H NMR spectrum of agrocybin I (**2**) (400 MHz, acetone-d_6)

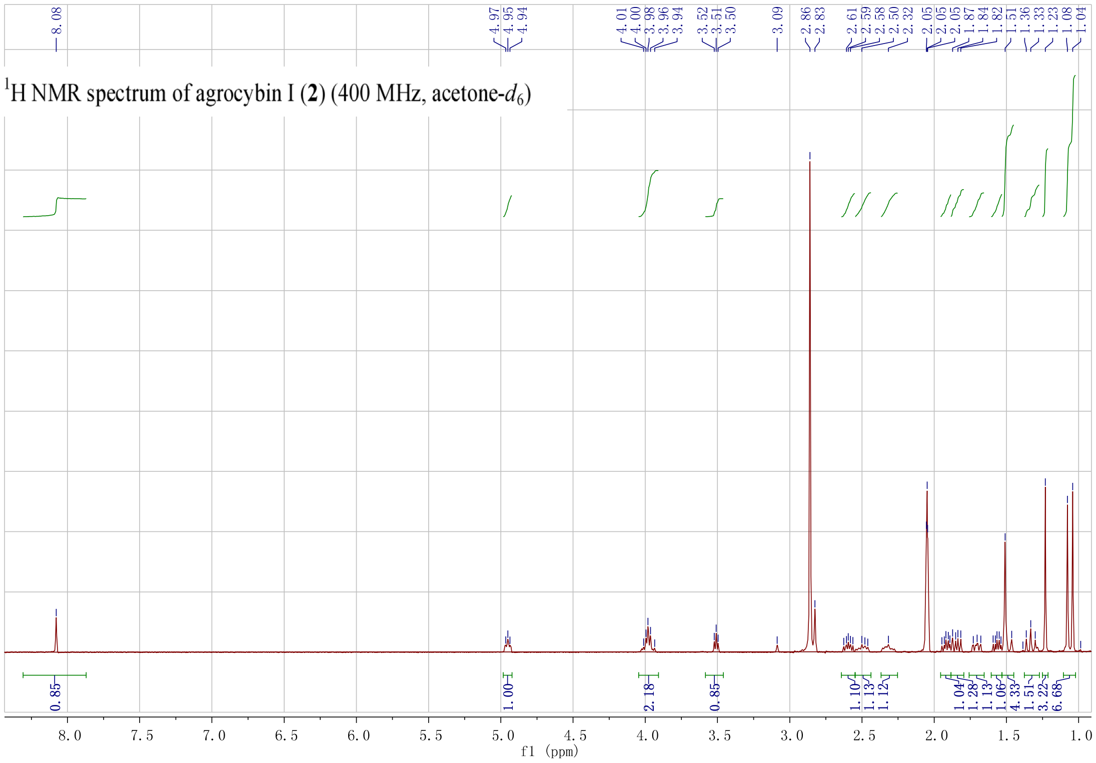

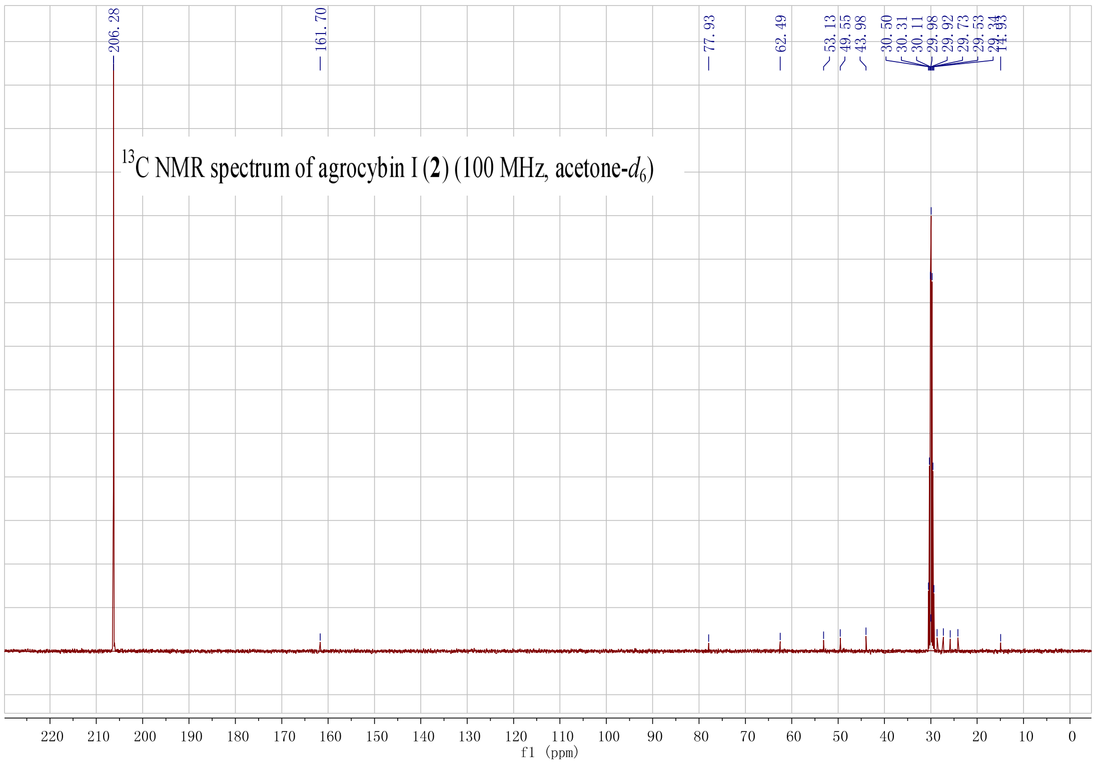

^{13}C NMR spectrum of agrocybin I (**2**) (100 MHz, acetone-d_6)

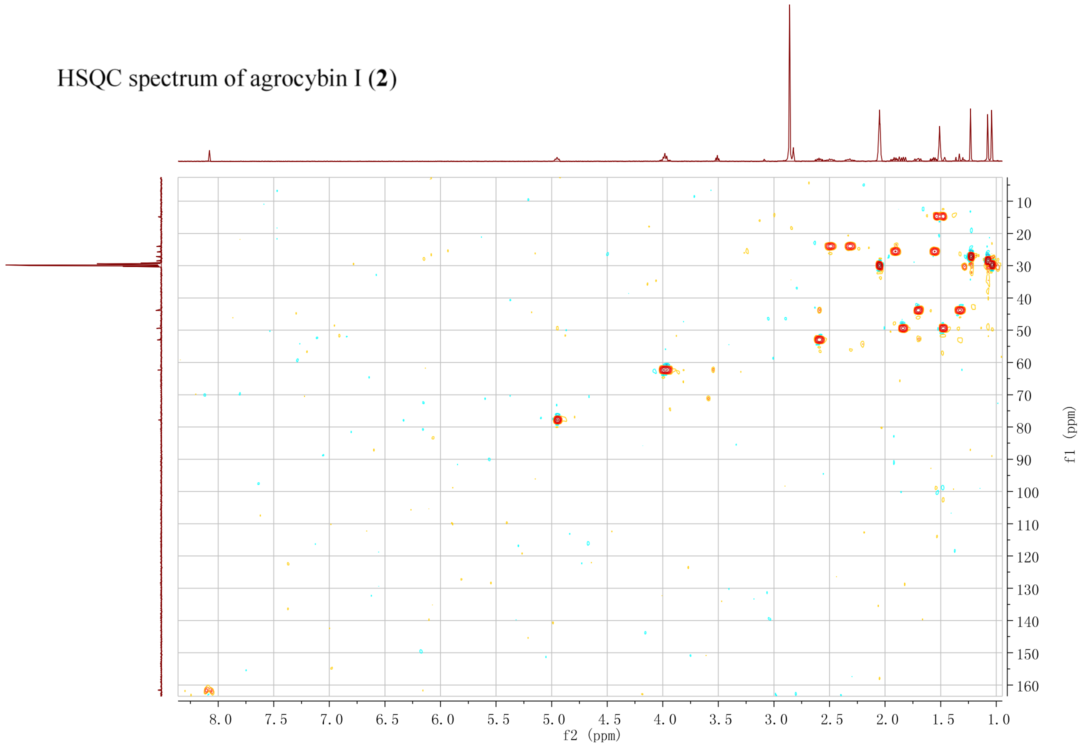

HSQC spectrum of agrocybin I (**2**)

HMBC spectrum of agrocybin I (**2**)

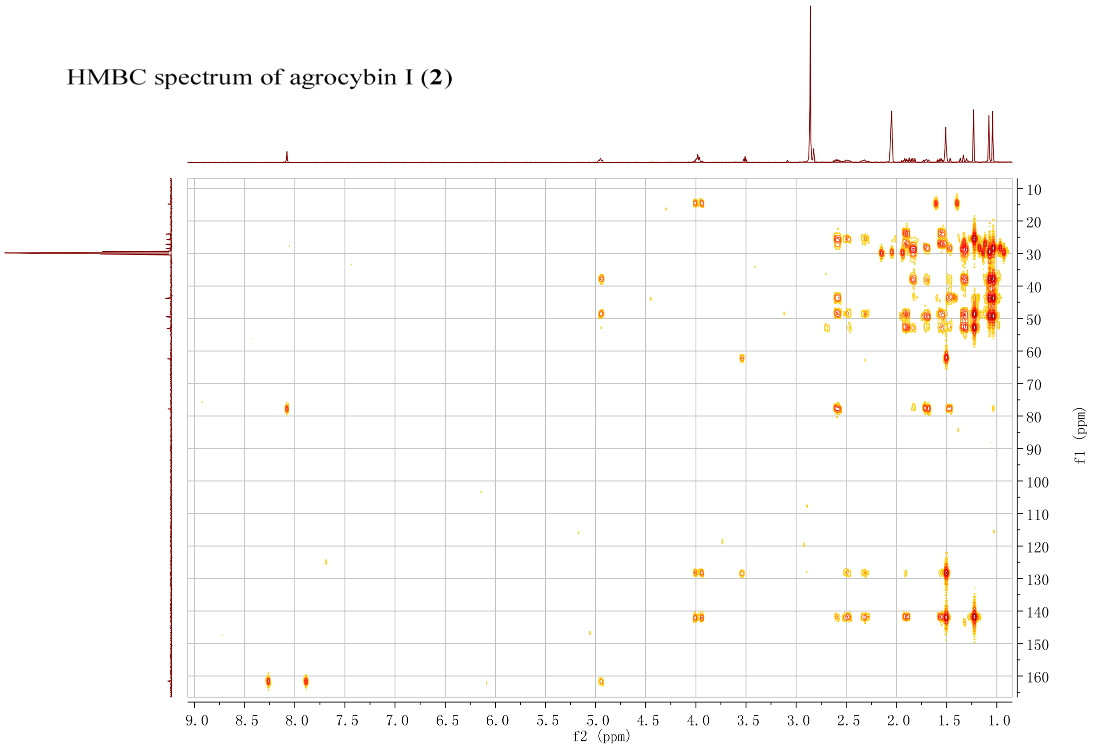

Supplement: Supplementary file 1 — Supplementary material, approximately 864 KB. [file 13659_2012_31_MOESM1_ESM.pdf]
